# Genome-wide analysis of cold imbibition stress in soybean, *Glycine max*

**DOI:** 10.3389/fpls.2023.1221644

**Published:** 2023-08-21

**Authors:** Siwar Haidar, Simon Lackey, Martin Charette, Mohsen Yoosefzadeh-Najafabadi, A. Claire Gahagan, Thomas Hotte, Francois Belzile, Istvan Rajcan, Ashkan Golshani, Malcolm J. Morrison, Elroy R. Cober, Bahram Samanfar

**Affiliations:** ^1^ Agriculture and Agri-Food Canada, Ottawa Research and Development Centre, Ottawa, ON, Canada; ^2^ Department of Biology, Ottawa Institute of Systems Biology, Carleton University, Ottawa, ON, Canada; ^3^ Department of Plant Agriculture, University of Guelph, Guelph, ON, Canada; ^4^ Department of Phytology, Institut de Biologie Intégrative et des Systèmes (IBIS), Université de Laval, Quebec City, QC, Canada

**Keywords:** soybean, imbibition, germination, GWAS, cold stress

## Abstract

In Canada, the length of the frost-free season necessitates planting crops as early as possible to ensure that the plants have enough time to reach full maturity before they are harvested. Early planting carries inherent risks of cold water imbibition (specifically less than 4°C) affecting seed germination. A marker dataset developed for a previously identified Canadian soybean GWAS panel was leveraged to investigate the effect of cold water imbibition on germination. Seed from a panel of 137 soybean elite cultivars, grown in the field at Ottawa, ON, over three years, were placed on filter paper in petri dishes and allowed to imbibe water for 16 hours at either 4°C or 20°C prior to being transferred to a constant 20°C. Observations on seed germination, defined as the presence of a 1 cm radicle, were done from day two to seven. A three-parameter exponential rise to a maximum equation (3PERM) was fitted to estimate germination, time to the one-half maximum germination, and germination uniformity for each cultivar. Genotype-by-sequencing was used to identify SNPs in 137 soybean lines, and using genome-wide association studies (GWAS - rMVP R package, with GLM, MLM, and FarmCPU as methods), haplotype block analysis, and assumed linkage blocks of ±100 kbp, a threshold for significance was established using the qvalue package in R, and five significant SNPs were identified on chromosomes 1, 3, 4, 6, and 13 for maximum germination after cold water imbibition. Percent of phenotypic variance explained (PVE) and allele substitution effect (ASE) eliminated two of the five candidate SNPs, leaving three QTL regions on chromosomes 3, 6, and 13 (Chr3-3419152, Chr6-5098454, and Chr13-29649544). Based on the gene ontology (GO) enrichment analysis, 14 candidate genes whose function is predicted to include germination and cold tolerance related pathways were identified as candidate genes. The identified QTLs can be used to select future soybean cultivars tolerant to cold water imbibition and mitigate risks associated with early soybean planting.

## 1 Introduction

Soybean, *Glycine max* (L.) Merr., has become one of the most important globalized commodities of the modern era. Canada has a role to play in the soybean economy, with over 2.1 million hectares (5.2 million acres) planted in 2022 across five principal growing regions (Soy Canada, 2023). Cold stress tolerance during different stages of development has limited the expansion of short-season soybean in Canada. Soybean cultivars tolerant to cold temperatures (15/5°C day/night) during flowering had 8% greater seed yield than susceptible cultivars (Cober et al., 2013). Alleles related to pubescence color have been shown to control seed coat discoloration induced by cool (<15°C) temperatures during soybean seed development (Morrison et al., 1998; Yamaguchi et al., 2021).

Germination is the first growth stage where the seed swells, sprouts, and becomes a seedling by expanding the radicle (Nonogaki et al., 2010). Soybean germination starts with the seed absorbing water, known as imbibition, which causes the seed coat to crack and the embryonic root to emerge (Bareke, 2018). During germination, the seed’s reserves are broken down to provide energy for growth. This process is regulated by hormones such as gibberellic acid (GA) and abscisic acid (ABA), which promote and inhibit germination, respectively (Miransari and Smith, 2014). Germination is a vital stage in the life cycle of a soybean plant, as it plays a crucial role in the early establishment of the crop and ultimately impacts yield. Several factors can influence the germination process, including but not limited to temperature, water availability, and the presence of pathogens. This process is further shaped by various ecological cues such as light, oxygen, and phytohormones produced endogenously by the seed (Shu et al., 2015; Oracz and Karpiński, 2016).

Research has demonstrated that temperature plays a significant role in the germination success rate and establishment of seedlings (Wuebker et al., 2001). Soybean are considered a warm season crop and require a minimum soil temperature of 10°C to germinate (Miransari and Smith, 2014). In Western Canada, the average soil temperature in the spring can vary widely, with temperatures often remaining below the minimum required for germination until well into the frost-free period. This can lead to delayed planting and reduced crop yields. Hypocotyl elongation was significantly delayed at 10°C and reached a maximum at 30°C (Hatfield et al., 1974). Alm et al. (1993) showed that as temperature increased from 10°C to 25°C, the soybean hypocotyl elongation rate increased. Optimal soybean growth and production has been well adapted to a range of environmental conditions, including soil temperatures ranging from 24°C to 40°C, with 28°C being the most optimal (Tyagi and Tripathi, 1983).

Plant hormones, such as GA and ABA, play a crucial role in regulating the germination process. GA produced in the embryo promotes germination by stimulating enzymes which digest the seed’s reserves in the cotyledons. In addition, they activate the growth of the embryonic root and shoot. On the other hand, ABA acts as a growth inhibitor and is responsible for maintaining the seeds dormant state. The balance of these hormones can significantly impact the rate and success of germination (Miransari and Smith, 2014). Auxin is another phytohormone that stimulates ABA signaling, thus, indirectly controlling seed dormancy (Shuai et al., 2017). Furthermore, the phytohormone jasmonic acid (JA) and its derivative methyl-jasmonate (Me-JA), found in soybean, have been shown to play different roles in some plants, including but not limited to similar effects as ABA (Yamane et al., 1981; Ueda and Kato, 1982b). JA can aid in the inhibition of germination (Koda, 1992), while Me-JA stimulates the synthesis of alkaloids such as diterpenoids and terpenoids (Yuanxin et al., 2013). The synthesis of terpenoids through the mevalonate pathway plays a role in plant development, including seed germination (Liao et al., 2014a). Additionally, seed coats that are impermeable to water and gases delay germination. Thus, scarification is a naturally occurring process that weakens the coat, increasing permeability to water and ultimately enhancing the germination rate (Priyadharshini and Lekha, 2021).

Marker trait association (MTA) analysis is a statistical approach that aims to identify the genetic markers associated with the expression of a trait of interest in a population. By analyzing the correlation between marker genotype and phenotype, MTAs can be utilized to identify potential candidate genes or genomic regions that are involved in the regulation of a specific trait. This type of analysis can provide insight into the genetic basis of a trait and its associated regulatory mechanisms (Huang and Han, 2014). Detecting MTAs associated with temperature-related seed germination traits in soybean is crucial for several reasons. Firstly, these traits are essential for early growth and plant establishment, which can influence crop productivity and yield (Schmutz et al., 2010). Secondly, identifying MTAs can provide valuable insights into the genetic mechanisms that regulate these traits, leading to the development of molecular markers and breeding strategies that enhance selection efficiency and precision. Finally, understanding the genetic basis of these traits can facilitate the identification of candidate genes and pathways involved in the plants response to environmental stress, allowing for the development of novel strategies to improve crop adaptability to changing environmental conditions (Hou et al., 2011).

One of the main limitations of linkage QTL mapping is the relatively low resolution it provides when identifying candidate genes. To overcome this limitation, genome-wide association studies (GWAS) have become widely used and a highly preferred method for identifying genomic loci associated with specific traits. GWAS is a cost and time-efficient approach that utilizes panels of theoretically unrelated genotypes and advanced bioinformatics tools to achieve high mapping resolutions (Brachi et al., 2011; Gupta et al., 2019; Ferreira and Marcelino-Guimarães, 2022). This approach has been widely adopted in many crop species including soybean (*G. max*) (Priyanatha et al., 2022). In soybean, GWAS has been used to study a wide range of agronomic traits, including resistance to soybean cyst nematode (Han et al., 2015; Vuong et al., 2015), yield and its components (Yoosefzadeh-Najafabadi et al., 2021), and responses to environmental conditions such as drought (Liu et al., 2020b; Saleem et al., 2022), and salinity (Zeng et al., 2017; Do et al., 2019). A short-season Canadian soybean GWAS panel that previously had been used to study agronomic traits such as maturity, plant height, seed weight, oil, protein, flower color, pubescence color, and hilum color (Sonah et al., 2015) will be used again in this study. The primary purpose of this study is to investigate the effect of cold water imbibition on soybean germination as early planting often exposes soybean seeds to cold water. Understanding the genetic factors influencing the germination process under such conditions is crucial. Limited research has been done on the tolerance to low temperatures in soybean. Most of the studies investigating the relationship between temperature and germination have employed linkage mapping and biparental populations (Liu et al., 2020a) rather than GWAS. Nguyen et al. (2021) explored the impact of different temperature regimes and waterlogging conditions, concluding that differences in hypoxia tolerance may significantly modify the germination phenotype. Additionally, Zhang et al. (2012) developed backcross inbred lines that differed in their germination rate between cold and warm treatments, but that study imbibed the seed at a constant and favorable temperature. We utilized a comprehensive marker dataset from Sonah et al. (2015) to perform genome-wide association analysis and haplotype block analysis, aiming to identify significant SNPs linked to maximum germination following cold water imbibition. Ultimately, the goal is to highlight key QTL regions and candidate genes related to germination and cold imbibition tolerance, which could be used for the selection of future elite soybean breeding cultivars. This research seeks to provide strategies to mitigate the risks associated with early soybean planting, thus contributing to the improvement of crop yield and sustainability under Canadian climatic conditions.

## 2 Materials and methods

Phenotypic assessment of resistance to cold imbibition stress was carried out on a panel of 137 short season Canadian soybean lines by subjecting seed to cold and warm imbibition treatments. Data was fitted to a time series then used in GWAS analysis to identify regions of interest and select candidate genes that play a role in contributing to meaningful phenotypic variance through beneficial alleles.

### 2.1 Selection of original GWAS panel

As previously reported in Sonah et al. (2015), 304 soybean elite cultivars were selected from maturity groups 000 to II, representing the existing diversity employed by Canadian soybean breeders. The population structure was used to identify 139 cultivars with the greatest genetic diversity, which were subjected to phenotyping for qualitative and agronomic traits (Sonah et al., 2015). Of these 139 genotypes, 137 were chosen for further investigation in this study based on the availability of their genotypic and phenotypic data.

### 2.2 Experimental design and phenotypic data collection

Soybean cultivars were grown in the field at Ottawa Research and Development Center in 2017, 2018 and 2021 in an unreplicated modified augmented design trial with replicated check cultivars. Plots were 3m long and 1.6m wide, each containing four rows. Planting dates ranged from May 24^th^ to June 2^nd^ and harvest dates ranged from September 21^st^ to October 6^th^. Seeds were stored in a refrigerator for 4 to 6 months after being cleaned until the start of the experiment. The experiment used four samples from each field plot for the 2017 and 2018 seed, and three samples for 2021 seed. Twenty seeds per sample from each cultivar were selected for structural integrity prior to being placed on filter paper in a 9 cm petri dish and subjected to either cold or warm imbibition. Both temperature treatments were initiated at the same time. Cold treated seeds had 10 mL of refrigerated water put onto the filter paper and were then placed in the refrigerator (4°C) overnight (16 hours) before being transferred to the warm germination cabinet (20°C). Warm treated seeds had 10 mL of water from the germination cabinet put onto the filter paper prior to being placed into the cabinet. Seeds were considered to be germinated when the radical was 1 cm in length and the germinated seed was removed from the dish. Observations were made daily from day two to seven after the first 10 mL of distilled water was added. Petri dishes were watered when needed with additional water at the germination cabinet temperature.

### 2.3 Calculation of three-parameter exponential rise to a maximum equation

A three-parameter exponential rise to a maximum (3PERM) equation was created according to Rooney et al. (2022) with some modifications. After phenotyping experiments were completed, a time series approach was used to calculate the phenotypic values used in the GWAS analysis. The following formula was used:


$$germination=\frac{A}{1+{\left(time/H\right)}^{B}}$$

where A is the predicted maximum germination, H is the time to one-half maximum germination (hours), and B is the slope of the line representing uniformity in germination. For this experiment, germination observations were concluded after 168 hours.

Nonlinear regression (Proc NLIN, SAS Institute Inc, Cary, USA) was used to determine A, H, and B, with initial parameters set at A = 95, H = 35, and B = -6 and A constrained to A ≤ 100% germination. To adjust for different germination potential for each seed sample, the cold A value was adjusted using the warm A value: cold maximum germination/warm maximum germination x 100. This new determined trait was referred to as “A Cold Adjusted.”

For each seed production year, mean values for each parameter were determined as well as the mean over all three years. A total of seven germination parameters were used in the GWAS analysis: A cold, A warm, A cold adjusted, B cold, B warm, H cold, and H warm.

### 2.4 GWAS analysis

A publicly available dataset from Sonah et al. (2015) was utilized for the GWAS analysis which was done in R software (R Core Team, 2021) using the rMVP package (Yin et al., 2021). To ensure accuracy and minimize the false-positivity rate, three models were utilized independently: General Linear Model (GLM), Mixed Linear Model (MLM), and Fixed and Random Model Circulating Probability Unification (FarmCPU) (Kusmec and Schnable, 2018). GLMs are a class of models that extend linear regression to handle non-normal distribution of data, such as binary or count data. MLMs, on the other hand, are models that account for both fixed and random effects in a dataset. FarmCPU is a method that employs MLMs to identify genetic associations between traits and markers in genomic data. This model uses a compressed MLM to improve computational efficiency and allow for analysis of large datasets (Yoosefzadeh-Najafabadi et al., 2022). The population structure was captured using the covariate P obtained from principle component analysis (PCA), and the relatedness between individuals was estimated using a kinship matrix (K) (VanRaden, 2008). The p-value cut-off for the statistical significance of SNPs was calculated using *qvalue* package in R based on the false discovery rate (Storey et al., 2022).

Calculation of allele substitution effect (ASE) was carried out to examine significant SNPs. In brief, cultivars were classified by alleles at each SNP independently, then the mean phenotypic value of contrasting classes of SNPs were determined. This showed the effect of changing the allele on the phenotype under study in the original units reported (Kato et al., 2021). A second method was applied using a linear regression approach to calculate R^2^ and the percent variance explained (PVE) by each SNP using the *lm()* function in R (R Core Team, 2021) as in Saleem et al. (2022).

### 2.5 Haplotype analysis

Linkage disequilibrium (LD) is a measure of the association between different loci on a chromosome. A 100 kbp assumed LD decay window upstream and downstream of significant SNPs was used to identify potential candidate genes using SoyBase (https://www.soybase.org) (Grant et al., 2010). Haplotype block analysis was carried out using Haploview 4.1 (Broad Institute, 2008). Hardy-Weinberg p-value cut-off was set to 0, and the solid spine method of LD block analysis was used with default parameters to generate haplotype blocks (Barrett et al., 2004).

### 2.6 Candidate gene identification

Genes within QTL regions were retrieved then over-under analysis was carried out using the SoyBase GO Term Enrichment Tool (Grant et al., 2010). The GO enrichment analysis tool was used to identify over-represented genes within the regions of significant SNPs obtained from GWAS and filtered using ±100 kbp LD decay distance, haplotype block analysis, ASE and PVE. Existing literature was used to verify the relevance of candidate genes and whether their annotations related to the germination process or cold tolerance.

## 3 Results

### 3.1 Time series germination parameters estimated using 3PERM

**Figure 1:**
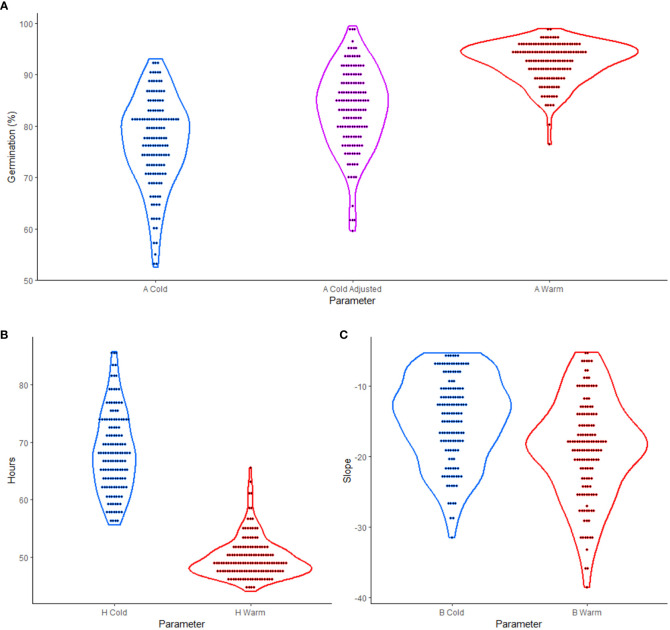
Violin plots of three-parameter exponential rise to a maximum (3PERM) in soybean cold and warm imbibition treatments. **(A)** A parameter showing percent germination for cold, cold adjusted, and warm treatments. **(B)** H parameter showing the time in hours to one half of the maximum germination for cold and warm treatments. **(C)** B parameter showing the slope for which more negative values represent more uniform germination in cold and warm treatments. In each case the dots represent over year mean values for the parameter for each genotype.

The experiment tested the effects of cold versus warm imbibition on germination using 137 soybean cultivars previously studied by Sonah et al. (2015). By employing a 3PERM equation to fit the time series germination data (**Supplementary Table 1**), germination parameters were estimated. The visual representation of over-year means of these parameters, as shown in **Figure 1**, revealed the variability in phenotypic data between cultivars. This observation provided a broad overview of the diverse responses in germination performance across different cultivars, supporting the further analyses and interpretation of the overall data trend.

### 3.2 GWAS analysis

**Figure 2:**
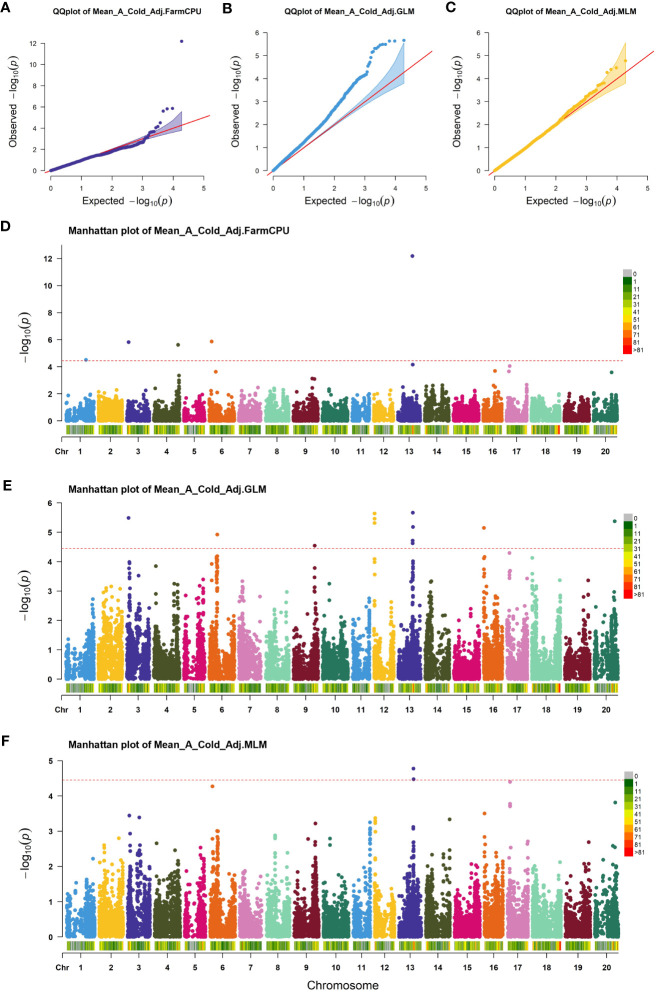
Association analysis of cold imbibition of soybean. **(A–C)** Quantile-quantile plot of FarmCPU, GLM, and MLM, respectively. **(D–F)** Manhattan plots of significant SNPs identified in FarmCPU, GLM, and MLM of GWAS analysis, respectively. The red horizontal line shows the threshold used for significance. Each colored dot indicates an associated SNP.

GWAS was performed on germination parameters estimated using a 3PERM equation fit to the time series phenotypic data and Genotyping-by-Sequencing (GBS) SNP data containing 155,617 SNPs across all 20 chromosomes. Three statistical models were chosen for our analysis, namely GLM, MLM, and FarmCPU. The choice of these models was informed by their unique strengths in addressing different complexities associated with genetic analyses. GLM is an effective tool for analyzing simple population structures, whereas MLM provides a more robust framework for handling complex kinship and population structures, potentially reducing false-positive associations (Balding, 2006; Kang et al., 2008). FarmCPU, on the other hand, takes a novel approach by implementing both fixed and random effects, enabling us to handle confounding effects of cryptic relatedness and population stratification simultaneously, thereby providing a more accurate mapping of quantitative trait loci (Liu et al., 2016). Our comparative analysis revealed subtle differences in the outcomes from all three models, with FarmCPU showcasing a lower false discovery rate and better statistical power, followed closely by MLM and then GLM. Considering these results, we focused on the FarmCPU model as it demonstrated the highest efficacy for our study, reducing spurious associations while maximizing the chances of detecting true associations, resulting in a more reliable and comprehensive set of marker trait associations. A Cold Adjusted was the focus of this study because it represented the phenotype in the most direct way. Five significant SNPs for A Cold Adjusted were identified using the FarmCPU model (**Figure 2**) located independently across five chromosomes (**Table 1**). In total, 90 genes, within ±100 kbp of each SNP, were identified using the SoyBase W82 Genome Browser (Grant et al., 2010). Nine genes, 15 genes, 22 genes, 27 genes, and 17 genes are found in the region of Chr1-40636558, Chr3-3419152, Chr4-49314680, Chr6-5098454, and Chr13-29649544, respectively. The list of genes is found in **Supplementary Table 2**.

**Table 1:** Significant SNPs identified in GWAS using FarmCPU.

QTL	Position	p-Value	-log10	Genes
**Chr1-40636558**	40636558	3.15E-05	4.502047565	9
**Chr3-3419152**	3419152	1.50E-06	5.823259179	15
**Chr4-4931468**	49314680	2.43E-06	5.613731176	22
**Chr6-5098454**	5098454	1.36E-06	5.865154533	27
**Chr13-2964954**	29649544	6.52E-13	12.18545814	17

Name of QTL, SNP position, p-value determined using FarmCPU, -log10 of P-value, and number of genes in the ±100 kbp region.

### 3.3 Haplotype analysis

**Figure 3:**
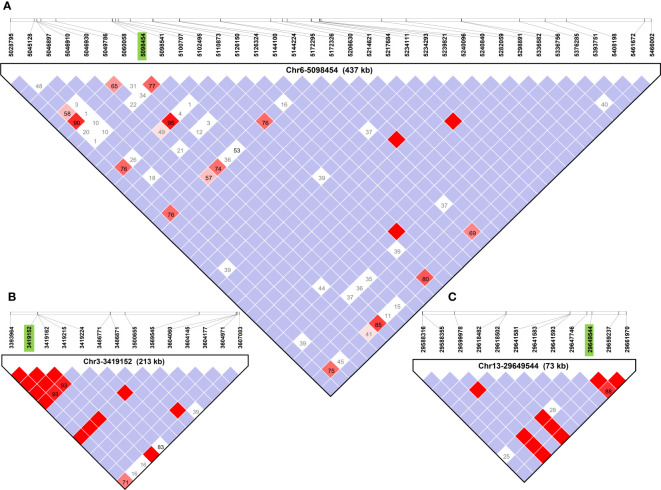
Haplotype blocks defined using Haploview 4.1 software for each QTL region identified in GWAS using FarmCPU for soybean. The white squares indicate logarithm of odds (LOD)<2, and normalized coefficient of linkage disequilibrium (D’) >1, suggesting little evidence of linkage between markers. Shades of pink and red indicate LOD ≤ 2 and D’>1 indicate some evidence of linkage, bright red indicates LOD ≤ 2 and D’=1 indicate stronger evidence of linkage, and blue indicates LOD<2 and D’=1 meaning that there is no evidence of linkage between markers. Boxes contain D’ values unless they are equal to 1 in which case they are omitted. **(A–C)** Haplotype blocks containing primary SNPs (in green) of Chr6-5098454, and Chr3-3419152, and Chr13-29649544, respectively. Haplotype blocks containing Chr1-40636558 and Chr4-49314680 are available in **Supplementary Figure 1**.

Haplotype block analysis added a total of 53 genes that were outside of the original ±100 kbp window, and these were retrieved using SoyBase W82 Genome Browser (Wm82.a2) (Grant et al., 2010) as shown in **Figure 3**. Thirteen, four and 36 genes were added for SNPs Chr1-40636558, Chr3-3419152, and Chr6-5098454, respectively (**Table 2**). Chr4-49314680 and Chr13-29649544 had haplotype blocks within ±100 kbp, so no new genes were included as candidates. The complete list of genes is found in **Supplementary Table 3**.

**Table 2:** Haplotype blocks containing significant SNPs as determined using Haploview.

SNP	Position	Start	End	Haplotype Block Size (kbp)	Total Genes	New Genes
**Chr1-40636558**	40636558	40406845	40903021	496.2	22	13
**Chr3-3419152**	3419152	3393964	3607083	213.1	14	4
**Chr4-49314680**	49314680	49297805	49332333	34.5	6	0
**Chr6-5098454**	5098454	5028795	5466002	437.2	58	36
**Chr13-29649544**	29649544	29588316	29661970	73.7	6	0

Size of each block, total genes located within the block and number of new genes not previously included in the ±100 kbp LD decay windows.

### 3.4 Allele substitution effect and percent variance explained

**Figure 4:**
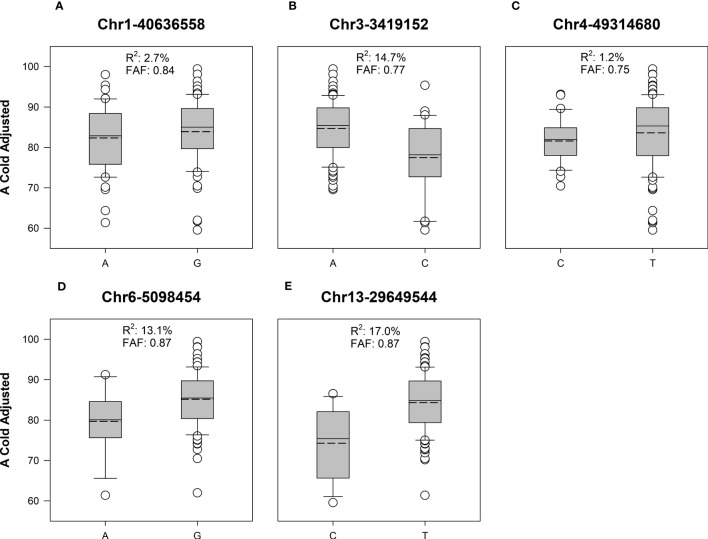
Box and whisker plots of adjusted cold germination between favorable and alternate alleles of significant SNPs in soybean cultivars. Solid line represents the median value, dashed line is the mean. Boxes include data points between the first and third quartile, whiskers represent the 5^th^ and 95^th^ percentile. Favorable allele frequency **(FAF)** represents the proportion of lines with the allele producing the highest value for A Cold Adjusted. **(A)** In Chr1-40636558, allele G is the favorable allele with higher A cold adjusted germination rate. **(B)** In Chr3-3419152, allele A is the favorable allele. **(C)** In Chr4-49314680, allele T is the favorable allele. **(D)** In Chr6-5098454, allele G is the favorable allele. **(E)** In Chr13-29649544, allele T is the favorable allele. In all cases the favorable alleles had a higher frequency than the alternate allele in the studied population.

ASE and PVE were calculated (**Table 3**). ASE effects for Chr1-40636558 and Chr4-49314680 were much lower than other SNPs. Similarly, the PVE for Chr1-40636558 and Chr4-49314680 was lower than other SNPs identified (**Figure 4**). These calculations show that significant marker trait associations do not always provide relevant changes to the phenotype and that the alleles present in our population may be of low effect.

**Table 3:** Allele substitution effect (ASE) and percent variance explained (PVE) for significant SNPs identified in GWAS for soybean cold imbibition.

SNP	Favorable Allele	Favorable Mean (% Germination)	Alternate Allele	Alternate Mean (% Germination)	Favorable Allele Frequency	ASE	R^2^	PVE
**Chr1-40636558**	G	83.92625	A	82.35543	0.84	1.57081	0.02715	2.71485
**Chr3-3419152**	A	84.6818	C	77.50512	0.77	7.17668	0.1472	14.7195
**Chr4-49314680**	T	83.60166	C	81.60276	0.75	1.9989	0.01166	1.16585
**Chr6-5098454**	G	85.14689	A	79.67438	0.87	5.47251	0.13053	13.0533
**Chr13-29649544**	T	84.30835	C	74.27877	0.87	10.0296	0.17034	17.0343

For each chromosome, the A Cold Adjusted mean value for favorable and alternate alleles are presented, as well as ASE, R^2^, and PVE.

### 3.5 Candidate gene identification

After the elimination of Chr1-40636558 and Chr4-49314680, since their effects were an order of magnitude less than the other three SNPs, 102 genes were identified using ±100 kbp linkage blocks, and haplotype block analysis in the regions of Chr3-3419152, Chr6-5098454, and Chr13-29649544. The results were pooled and analyzed based on GO terms using the SoyBase GO Term Enrichment Tool (Grant et al., 2010), resulting in a shortlist of 14 candidate genes. Genes were chosen based on their gene ontology terms relating to germination and the number of genes found to be over-represented (**Table 4**).

**Table 4:** Candidate genes for SNPs on chromosomes 3, 6, and 13 whose over-represented GO terms are related to germination pathways in *Glycine max*.

Gene Name		GO	GO Description
*Glyma.13g183600*		GO:0009695	jasmonic acid biosynthetic process
		GO:0010333	terpene synthase activity
		GO:0016102	diterpenoid biosynthetic process
*Glyma.06g068600*		GO:0006833	water transport
		GO:0006972	hyperosmotic response
		GO:0009266	response to temperature stimulus
		GO:0009789	positive regulation of abscisic acid mediated signaling pathway
		GO:0009845	seed germination
*Glyma.06g068700*		GO:0006833	water transport
		GO:0006972	hyperosmotic response
		GO:0009266	response to temperature stimulus
		GO:0009789	positive regulation of abscisic acid mediated signaling pathway
		GO:0009845	seed germination
*Glyma.06g069600*		GO:0006833	water transport
		GO:0006972	hyperosmotic response
		GO:0009266	response to temperature stimulus
*Glyma.03g030600* *Glyma.03g030800* *Glyma.03g030900* *Glyma.03g031000* *Glyma.03g031300* *Glyma.03g031400*	Cytochrome p450	GO:0000096	sulfur amino acid metabolic process
		GO:0006569	tryptophan catabolic process
		GO:0009695	jasmonic acid biosynthetic process
		GO:0009695	jasmonic acid biosynthetic process
		GO:0019288	isopentenyl diphosphate biosynthetic process, mevalonate-independent pathway
		GO:0044272	sulfur compound biosynthetic process
		GO:0052544	defense response by callose deposition in the cell wall
*Glyma.03g031800*		GO:0010492	maintenance of shoot apical meristem identity
*Glyma.06g071500*		GO:0009266	response to temperature stimulus
*Glyma.06g066200*		GO:0010072	primary shoot apical meristem specification
*Glyma.06g071300*		GO:0016114	terpenoid biosynthetic process

GO descriptions retrieved from SoyBase Go Term Enrichment Tool.

## 4 Discussion

Measuring cold imbibition stress in the field is a challenging task due to the confounding effects of variable germination rates, microclimates, field conditions, and the absence of ways to induce the stress on a large scale. Furthermore, the labor and time required to count emerging seedlings further complicates the situation. In this study, to overcome these challenges, we developed an indoor assay for the phenotype. Germinating seeds in refrigerated water was used to simulate cold imbibition stress and can be done in a reproducible manner to ensure that findings are consistent over years. Additionally, this method allows for the replication of experiments if new germplasm needs to be tested to identify rare alleles.

It is critical to note that soybean seeds undergoing imbibition are at high risk of injury following exposure to sub-optimal temperatures, particularly cold. Dehydration of seeds during the maturation process causes membranes to change from lamellar organization to hexagonal II organization (Steponkus and Lynch, 1989). The seed begins the process of recovery from its dormant state with imbibition; membranes re-organize and regain their function allowing cellular processes to resume (Suo et al., 2022). The reduced overall fitness associated with delays in plasma membrane reorganization can delay the advancement of the germination process and negatively impact seedling establishment (Leopold, 1980).

Furthermore, the decreased efficiency of the plasma membrane is due to the altered reaction rate of membrane-bound proteins, ion leakage, and a loss in compartmentalization (Bhattacharya, 2022). Genotypes showing a reduction in leakage of cellular contents during cold imbibitional injury will achieve a higher germination rate and have better seedling establishment rates in poor weather conditions (Borowski and Michałek, 2014). The highest risk for imbibitional chilling injury occurs during the first 24 hours after planting, especially for seeds planted in cold soil, followed by a further reduction in soil temperature (Pollock and Toole, 1966). Understanding these processes guides our work in identifying genomic regions controlling contrasting response to cold imbibition stress and will contribute to the understanding of the germination process under this and other abiotic stresses.

QTLs Chr3-3419152, Chr6-5098454, and Chr13-29649544 are the most important regions identified in this association analysis based on the FarmCPU model using calculations of ASE, and PVE. Chr1-40636558 and Chr4-49314680 played a minor role in cold imbibition stress in this panel but were found to play a role in germination-related traits in other studies investigating low temperature tolerance and drought tolerance at the germination stage (Zhang et al., 2012; Zhao et al., 2022).

Consolidation of information gathered about each gene in QTL regions allowed the selection of genes based on those expected to be most involved in the regulation of cold imbibition. We propose the following as our top candidate genes for further study: *Glyma.13G183600, Glyma.06G068600, Glyma.06G068700*, and a cluster on chromosome 3 that includes the genes *Glyma.03G030600*, *Glyma.03G030800*, *Glyma.03G030900*, *Glyma.03G031000*, *Glyma.03G031300*, and *Glyma.03G031400*.

*Glyma.13G183600* has relevant gene ontology terms spanning several important germination-related pathways. The SNP associated with this gene was found to be significant across all three GWAS models applied (GLM, MLM, and FarmCPU). Other nearby SNPs on chromosome 13 show a similar allelic pattern and led us to believe that this is a region of interest in this study. Interestingly, this was the only candidate gene selected from the QTL on chromosome 13. It is located in the smallest region identified in this study and is the only plausible candidate in that region based on gene ontology annotations. Nine other SNPs are present in this QTL region, but none were found to be significant in the association analysis. *Glyma.13G183600* is orthologous to *terpene synthase 04* in *Arabidopsis thaliana* and has several over-represented gene ontology terms related to germination: jasmonic acid biosynthesis process, diterpenoid biosynthetic process, and terpene synthase activity.

*Glyma.06G068600* and *Glyma.06G068700* were included as candidates after carrying out haplotype block analysis for Chr6-5098454. This QTL region is the largest in the study, but the top candidates are located near the center of this QTL region, outside of the ±100 kbp window but well within the boundaries determined from haplotype block analysis. Gene ontology annotations of response to temperature stimulus and seed germination, as well as positive regulation of ABA-mediated signaling pathway, water transport, and hyperosmotic response, are all over-represented. Changes in water transport and hyperosmotic response could directly impact the rate of seed imbibition.

Finally, the cluster of genes located at the QTL region on chromosome 3 is related to cytochrome p450, including *Glyma.03G030600*, *Glyma.03G030800*, *Glyma.03G030900*, *Glyma.03G031000*, *Glyma.03G031300*, and *Glyma.03G031400*. This series of genes are all identical in terms of gene ontology annotations and are over-represented for tryptophan biosynthetic process, defense response by callose deposition in the cell wall, sulfur compound biosynthetic process, sulfur amino acid metabolic process, tryptophan catabolic process, jasmonic acid biosynthetic process, and isopentenyl diphosphate biosynthetic process (mevalonate-independent pathway). This gene family is very common, making up around 1% of the genome of different plant species (Mizutani, 2012). A mutation in a housekeeping gene could cause knock-on effects on many biological pathways relating to cold imbibition, germination, and temperature response, without completely disrupting the basic function of the processes due high copy number of related genes. This may be because the mutation may only affect a specific region or pathway within the gene, which may not be directly involved in the core functions of the housekeeping gene or could be due to redundancy in gene function, gene regulation, pleiotropy, or epigenetics. The region on chromosome 3 containing cytochrome p450 genes extends beyond the boundaries of the QTL region identified in this study.

Other studies have investigated the impact of abiotic stress on germination and germination-related parameters. Zhang et al. (2012) investigated the impact of temperature on germination, as in the present study; however, seed imbibition was carried out at a constant temperature followed by a period of cold treatment during the germination phase. Of the nine regions identified in Zhang et al. (2012), there are two in close proximity to those identified in this study. The first common QTL, identified by the Satt633 marker, is 3.8 Mbp downstream of the Chr13-29649544 from this study and had low favorable allele frequency with a high phenotypic effect. The Chr13-29649544 region in this study had a high favorable allele frequency and high phenotypic effect. The second common QTL, identified by the Satt338 marker, is only 678 kbp upstream of the region identified as Chr4-49314680 in our study. The favorable alleles at both QTLs on chromosome 4 are high in frequency but low in effect and their proximity suggests that they may reflect the same underlying causative genes. Tolerance to both cold stress during imbibition and following imbibition may be controlled by the same genomic regions.

Another set of important abiotic stresses being studied in relation to germination are drought and salinity. Zhao et al. (2022) studied drought tolerance through simulated drought conditions by varying osmotic stress during imbibition and germination and identified 26 SNPs using GWAS on a 410 accession diversity panel. Five of the 26 SNPs coincide with those identified herein: Gm01_38948188 is 2.0 Mbp downstream from Chr1-40636558, Gm03_39037 is 3.6 Mbp downstream from Chr3-3419152, Gm04_50945875 is 1.5 Mbp upstream from Chr4-49314680, Gm06_9791913 is 4.3 Mbp upstream from Chr6-5098454, and Gm13_35517964 is 5.8 Mbp upstream from Chr13-29649544. Liu et al. (2020b) used a simulated drought approach to investigate seed germination rates under drought stress, identifying 15 SNPs. One SNP linked to marker ss248097753 was 1.8 Mbp downstream from Chr13-29649544. Interestingly, Liu et al. (2020b) tied this SNP to one previously linked to oil/protein (Eskandari et al., 2013a; Eskandari et al., 2013b) from a recombinant inbred line (RIL) population with OAC Wallace as the donor of the favorable allele for oil. In our work, OAC Wallace also carried the favorable allele for cold imbibition tolerance, suggesting that all three groups identified an important locus that could impact multiple agronomic traits.

Shi et al. (2022) investigated the effect of salinity on germination of *Glycine soja* seedlings using a RIL population and GWAS to identify 25 QTLs, 21 significant SNPs, and 24 suggestive SNPs. The identified SNP on chromosome 3 at position 3141656 was linked to salt tolerant germination and is located in close proximity to our QTL region Chr3-3419152. Similarly, Kan et al. (2015) identified 13 SNPs related to salt tolerance and germination using *G. max*, including one 401 kbp downstream of our Chr3-3419152 region and very close to the one identified in Shi et al. (2022). This group of findings validates the results obtained in this GWAS and suggests that the region identified on chromosome 3 could have an important role in germination under both salinity and cold stress in both *G. max* and *G. soja* germplasm.

In contrast to this study where fresh seeds were used in developing phenotypic data, several studies have investigated the effects of both normal and artificial aging on germination parameters. Jiamtae et al. (2022) used a RIL population of untreated seeds and seeds treated at both 25°C and 35°C for six months to measure changes in germination. Five QTLs were identified on chromosomes 2, 6, and 8, with the one on chromosome 6 being most proximal to the one found in this study. Zhang et al. (2019) also identified 34 QTLs related to germination rate after various seed storage conditions. Notable from the Zhang et al. (2019) study was a hotspot on chromosome 3 in the region of the other chromosome 3 QTLs detected by Shi et al. (2022); Kan et al. (2015), and in the current study. This further supports the likelihood that the region detected on chromosome 3 is important in the germination process.

Alongside the aforementioned abiotic factors, the synthesis of phytohormones has a crucial role in seed germination. Plants control their functions through the synthesis of phytohormones in response to stress. ABA suppresses growth and preserves seed dormancy, while GA facilitates germination (Miransari and Smith, 2014). Moreover, JA and Me-JA are phytohormones found in many plants, including soybean, that are linked to germination and may have roles like the ABA pathway (Yamane et al., 1981; Ueda and Kato, 1982a). JA and Me-JA have been shown to inhibit seed germination in multiple plant species (Nojavan-Asghari and Ishizawa, 1998; Bogatek et al., 2002; Norastehnia et al., 2007; Ahmadi et al., 2014), and the synthesis of terpenoids through the mevalonate pathway plays a role in plant development, including seed germination (Wang et al, 2012; Liao et al., 2014b). Additionally, tryptophan inhibits germination and growth in some plant species, while its derivatives melatonin and serotonin increase during germination in soybeans (Nontasan et al., 2021). Isoprenoids, synthesized in the mevalonate-independent pathway, are linked to pea-seed germination (Green and Baisted, 1972; Rohmer et al., 1996).

Several possible limitations of this study include low sequencing depth of the original GWAS panel, false positive marker trait associations that can occur in GWAS analysis, and the reliance on emerged radicle observations as a proxy for germination. Recent GWAS studies are using datasets with increasing sequencing coverage thanks to continual improvements in cost and efficiency due to technological advancement. Scoring germination based on emerged radicle observations works well in most cases but does not account for other common germination factors such as soil type and seedling vigor. Seedlings with low vigor may produce a radicle but may lack the energy reserves that would allow it to push through compacted soil and achieve emergence.

In conclusion, our study identified three candidate regions Chr3-3419152, Chr6-5098454, and Chr13-29649544, containing genes proposed to be responsible for the difference in cold imbibition stress response: *Glyma.13G183600, Glyma.06G068600, Glyma.06G068700*, and the cluster on chromosome 3 that includes the genes *Glyma.03G030600*, *Glyma.03G030800*, *Glyma.03G030900*, *Glyma.03G031000*, *Glyma.03G031300*, and *Glyma.03G031400*. The genes are predicted to be involved in imbibition, germination, and temperature response pathways. Drawing on these findings, future work should focus on sequencing of candidate regions, gene expression analyses, or development of RILs using marker-assisted selection.

## Data availability statement

The datasets presented in this study can be found in online repositories. The names of the repository/repositories and accession number(s) can be found in the article/**Supplementary Material**.

## Author contributions

Conceptualization, BS, EC, MM; methodology BS, EC, MC, MM; formal analysis, SH, SL, EC, MY-N, MC, BS; investigation, BS, EC, MM, FB, SH, SL; data curation, MM, AG, TH; writing—original draft preparation, SH, SL; writing—review and editing, SH, SL, EC, AG, FB, IR, MY-N, BS, MM; supervision, MM, BS, AG, EC, IR; project administration, BS, EC, MM.; funding acquisition, BS, EC. All authors have read and agreed to the published version of the manuscript.
